# Complement anaphylatoxins as immune regulators in cancer

**DOI:** 10.1002/cam4.241

**Published:** 2014-04-08

**Authors:** Eli T Sayegh, Orin Bloch, Andrew T Parsa

**Affiliations:** Department of Neurological Surgery, Northwestern University Feinberg School of MedicineChicago, Illinois

**Keywords:** Anaphylatoxins, cancer, complement, immune, microenvironment

## Abstract

The role of the complement system in innate immunity is well characterized. However, a recent body of research implicates the complement anaphylatoxins C3a and C5a as insidious propagators of tumor growth and progression. It is now recognized that certain tumors elaborate C3a and C5a and that complement, as a mediator of chronic inflammation and regulator of immune function, may in fact foster rather than defend against tumor growth. A putative mechanism for this function is complement-mediated suppression of immune effector cells responsible for immunosurveillance within the tumor microenvironment. This paradigm accords with models of immune dysregulation, such as autoimmunity and infectious disease, which have defined a pathophysiological role for abnormal complement signaling. Several types of immune cells express the cognate receptors for the complement anaphylatoxins, C3aR and C5aR, and demonstrate functional modulation in response to complement stimulation. In turn, impairment of antitumor immunity has been intimately tied to tumor progression in animal models of cancer. In this article, the literature was systematically reviewed to identify studies that have characterized the effects of the complement anaphylatoxins on the composition and function of immune cells within the tumor microenvironment. The search identified six studies based upon models of lymphoma and ovarian, cervical, lung, breast, and mammary cancer, which collectively support the paradigm of complement as an immune regulator in the tumor microenvironment.

## Introduction

The tumor microenvironment represents a crucial context for understanding cancer 1, and is subject to varying levels of immunosurveillance and immunosuppression. Recent work has suggested that the complement anaphylatoxins C3a and C5a enhance tumor growth by shifting the balance toward immunosuppression 2, challenging longstanding dogma that complement activation is advantageous in cancer patients 3,4. Furthermore, the ability of neoplastic cells to evade attack by complement proteins while simultaneously activating complement undermines traditional concepts of complement in tumor control 5. Thus, in this article, the literature is reviewed in order to summarize the existing evidence in experimental cancer models on the potential role of complement as an immune regulator in the tumor microenvironment.

### The complement cascade

The complement cascade is an effector arm of innate immunity consisting of over 30 soluble and membrane-bound plasma proteins. This system, which evolved as a safeguard against nonself elements, is activated early in the immune response and is conventionally viewed as a mediator of cellular destruction. Complement can be activated through three pathways: classical, lectin, and alternative. The formation of C3 convertase is a shared step in all three pathways that is required for the generation of complement effectors 6, and in this process, the bioactive cleavage products known as anaphylatoxins are elaborated. Other key components of complement are C3b, an effector of opsonization and phagocytosis, and the membrane attack complex (MAC) formed by C5b-C9, a membrane pore-forming cytotoxic compound. Complement proteins are primarily synthesized in the liver and secondary lymphoid tissue, and subsequently circulate in the blood 7.

### Pathological complement signaling

The anaphylatoxins are potent chemoattractants and inflammatory mediators whose effects include smooth muscle contraction, histamine release from mast cells, promotion of vascular permeability, leukocyte chemotaxis, and elaboration of reactive oxygen species 8. Inappropriate complement activation and anaphylatoxin-mediated inflammation have been implicated in several pathological conditions in humans, including sepsis, neurodegenerative disease, autoimmune arthritis, ischemia-reperfusion injury, and spontaneous abortion 9. While the role of inflammation in cancer has historically been controversial, it has been tied to both tumor initiation and progression 1. According to this paradigm, complement anaphylatoxins may permit tumor growth by sustaining chronic inflammation 3,4, as these proteins are important regulators of the inflammatory response 10. It has been suggested that tumor-associated inflammation in humans tends to be chronic rather than acute, and thus may preferentially aid tumor promotion rather than tumor immune surveillance 1.

A myriad of diverse functions of complement are recognized aside from its opsonizing and cytolytic functions directed toward microbes. Complement is now recognized to mediate clearance of immune complexes and apoptotic cells 6, promotion of tissue regeneration 11, trafficking of hematopoietic progenitor cells 12, and angiogenesis 13. Beyond their role in inflammation, the anaphylatoxins appear to have nuanced roles in regulating adaptive immunity 8. While complement activation is highly regulated in physiological states, it is augmented under pathological conditions such as infection or tissue injury 8. Recent work has revealed that C3 and C5, the major constituents of this cascade, can be alternatively activated by a heterogeneous array of innate molecules to produce C3a and C5a 14,15. Although C5 activation typically requires the presence of activated C3, it can also independently occur in select pathological settings 15.

### The tumor microenvironment and its contribution to immune escape

The tumor microenvironment consists of multiplying tumor cells, stroma, associated tissue cells, blood vessels, and infiltrating inflammatory cells 16. Establishment of the tumor microenvironment is a stepwise process initiated by tumor hypoxia and ischemia 17 and subsequently marked by interstitial and cellular edema, a chronic inflammatory infiltrate, neovascularization, and tissue repair 16. The molecular correlates of this process include activation of the NF-*κβ* pathway and generation of reactive oxygen species (ROS), which promote local immunosuppression and secretion of proinflammatory cytokines like tumor necrosis factor (TNF)-*α* 16. While tumor cells upregulate major histocompatibility complex (MHC) class I polypeptide-related sequence A (MICA) and B (MICB) molecules and may cease to express human leukocyte antigens 18, seemingly making them more susceptible to innate immune recognition and attack, their predominant expression of “self” antigens makes them less immunogenic and explains the modest host reaction to neoplasms relative to bacterial and viral infections 19. Immunosuppressive cells in the tumor microenvironment further impede the host response by aiming, conceivably, to reverse this attack against “self.”^16^

The milieu of the tumor microenvironment is determined by the tumor, and facilitates tumor evasion of both innate and adaptive immunity. The tumor also recruits diverse subpopulations of immune effector cells and actively signals them toward not only a functionally suppressed but also tumor-promoting phenotype, or induces apoptosis of antitumor immune cells, in effect hijacking the local and systemic host defenses 16. At the same time, this inflammatory infiltrate represents host immune recognition of the abnormal, nascent tumor and an attempt to control it 16. Importantly, immunosuppression in cancer is a multifocal process wherein bone marrow homeostasis is also disrupted. As a result, irregular myelopoiesis and recruitment of myelomonocytic cells to the tumor and lymphoid tissue occur in synchrony with changes in the tumor microenvironment and peripheral immune centers 20–22. The complement anaphylatoxins have been implicated as tumor-elaborated signals that facilitate both the formation of this altered microenvironment and suppression of infiltrating immune cells 5.

Much of the attention directed toward complement in cancer has centered on membrane complement regulatory proteins (mCRPs), which represent another mechanism of tumor immune evasion. Tumor cells escape host complement defenses through overexpression of mCRPs 23. These factors are physiologically important for shielding host cells from complement-mediated attack 1. The immunoediting hypothesis suggests that this strategy developed in response to the selective pressure of complement activation within the tumor microenvironment 24. Similarly, tumor cells inactivate complement by secreting soluble complement inhibitors into the local microenvironment 25. mCRPs either act centrally at the level of C3 or terminally at the level of the MAC to prevent complement activation 1. The most commonly identified mCRPs in human cancers are CD46, CD55, and CD59 26–29. Importantly, mCRPs represent an obstacle to the efficacy of investigational cancer therapeutics, namely monoclonal antibodies (mAbs) directed against tumor-associated antigens with the intent of triggering antibody-dependent cell-mediated cytotoxicity (ADCC) and complement-dependent cytotoxicity 30.

### Immune effector cells within the tumor microenvironment

Effectors of adaptive immunity in the tumor microenvironment include CD4+ and CD8+ T cells, natural killer T (NKT) cells, dendritic cells, and infrequent B cells 16,31. Innate immune mediators permeating the tumor include M1-phenotype tumor-associated macrophages (TAMs) and sparse polymorphonuclear leukocytes and natural killer (NK) cells 16. Upon antigenic stimulation, dendritic cells (DCs) function as antigen-presenting cells (APCs) and play an integral role in orchestrating activation of naïve T cells. Although CD4+ and CD8+ T cells often form clonal populations with antigenic specificity for tumor cells, their ability to control the tumor is considerably diminished 16. DCs also have varied immunomodulatory functions such as induction of tolerance, determination of the T helper 1 (Th1) to T helper 2 (Th2) balance, and control of regulatory T (Treg) and T helper 17 (Th17) cell development 8. As polymorphonuclear leukocytes are more important to the acute than chronic inflammatory response, they are fairly rare in the tumor infiltrate, aside from collections of eosinophils in certain squamous cell tumors and granulocytes in various murine cancer models 16,32. The near absence of NK cells in the tumor microenvironment, in spite of their tumor cytotoxicity in vitro, may be a manifestation of tumor immune evasion 16.

A contrasting subset of immune cells allows tumor growth through suppression of the antitumor immune response. Chief among these are Treg cells 33, myeloid-derived suppressor cells (MDSCs) 21, and M2-phenotype TAMs 1. Tregs and MDSCs are extensively and consistently represented in the tumor inflammatory infiltrate, and are strongly associated with disease progression in several cancers 16. Tregs are a subtype of CD4+ CD25+ FoxP3+ T cells that are physiologically desirable for preventing autoimmunity. However, they are considerably expanded in the tumor inflammatory infiltrate relative to the peripheral blood 16. Acting through paracrine factors or contact-dependent mechanisms, Tregs prevent the proliferation of local T cells involved in the cell-mediated immune response 16. MDSCs are a variegated subset of CD11b+ Gr-1+ immature APCs with homology to macrophages and neutrophils, which pool in the bone marrow, peripheral blood, lymphoid tissue, and tumor microenvironment 34. Malignancies recruit MDSCs from the bone marrow to protect tumor cells from T cell-mediated host defenses. This process involves the production of highly suppressive ROS and reactive nitrogen species (RNS), and results in dysfunction of T cell-dependent tumor cytotoxicity in both animals and humans with cancer 21. While MDSCs are present at physiological levels even in individuals without cancer, their levels multiply in the blood of cancer patients and in the spleens of tumor-harboring mice 35. MDSC-mediated immunosuppression has been cited as the chief impediment to investigational cancer immunotherapies in clinical trials 36. M2-phenotype TAMs are interrelated immunosuppressive cells that inhibit the lymphocytic response and facilitate tumor progression. TAMs can be polarized toward an M2 phenotype when exposed to local MDSCs and tumor-derived factors and cytokines 16,37.

### Complement anaphylatoxins as immune regulators

Of late, the immunomodulatory properties of C3a and C5a and their receptors have been extensively characterized in the immunological literature, providing the basis for their investigation in cancer models. Several myeloid-derived innate immune cells express C3aR and C5aR, including monocytes, macrophages, DCs, neutrophils, basophils, mast cells, and eosinophils 38–49. Furthermore, their expression in these myeloid cells is regulated by complement components and inflammatory molecules 50–53.

T cells also express C3aR and C5aR 51,54–58. In turn, the anaphylatoxins regulate differentiation of T cells into different subsets, including Th1, Th2, Th17, and Treg, although contradictory roles have been observed in different studies 8,58–62. Acquisition of a particular phenotype in naïve T cells and subsequent travel to an inflammatory site 63 depend on the type of antigen and APC involved and the activation state 64. In this respect, complement may modulate T-cell activation either directly or indirectly by acting on APCs or toll-like receptors, including through cell surface deposition or exosomal release of complement activation products 64,65. In the setting of local complement activation, signaling through C3aR and C5aR may regulate antigen uptake, costimulation, and T-cell proliferation and differentiation 65.

An immunosuppressive role for C5aR signaling has been identified in models of allergic disorders involving pulmonary dendritic cells and Treg cells, where it dampens the immune response to inhaled antigens 66–68. C5a primarily appears to be a positive regulator of Th1 responses in models of infection, autoimmune disease, and organ transplantation, but a negative regulator in the context of parasitic infection and tumor growth 8,69. Through its influence on innate immune cells including DCs and macrophages, C3a similarly regulates the T cell response, especially the determination of Th1 cells 8. Both C3a and C5a have been implicated, albeit not consistently, in bolstering pathological Th2 immunity in conditions like asthma and dermatitis 70–73. C3aR and C5aR signaling also increases levels of the immunosuppressive cytokine interleukin (IL)-10 and reduces levels of IL-12 and interferon (IFN)-*γ*, which are important for T-cell differentiation 51,54,58.

## Methods

A systematic review of the English-language literature was performed. Articles were identified via PubMed search using Boolean operators and the key words “C3a,” “C5a,” “C3aR,” “C5aR,” and “complement” in combination with immune cells of interest involved in immunosurveillance (e.g., “monocyte*,” “macrophage*,” “dendritic cell*,” “lymphocyte*,” “Th1,” “Th2”) and immunosuppression cell (e.g., “Treg*,” “Th17,” “MDSC*”) as well as the general terms “immune,” “leukocyte*,” “immunosuppression,” and “microenvironment.” This search yielded 924 articles. These results were then individually reviewed to identify studies that investigated the immunomodulatory role of complement anaphylatoxin-mediated signaling within the tumor immune microenvironment. Six studies published between 2007 and 2012 were included in this review, which conducted in vitro, in vivo, and/or ex vivo investigations of this nature in models of ovarian cancer, lymphoma, lung cancer, mammary cancer, breast cancer, and cervical cancer (Tables1 and 2).

**Table 1:** Summary of evidence implicating the complement anaphylatoxins as regulators of MDSCs, Tregs, monocytes and macrophages, and NK cells in experimental cancer models

Study	Cancer Model	MDSCs	Tregs	Monocytes and macrophages	NK cells
Nunez et al. 74	Ovarian	Unchanged by C3 deficiency	Reduced by partial C3 deficiency	Macrophage levels unchanged by C3 deficiency	–
Gunn et al. 75	Lymphoma	Increased (splenic) by C5a overexpression; MDSCs from C5a (+) tumors were less immunosuppressive	C5a mediates Treg differentiation	Increased macrophage infiltration in C5a (+) tumors	Increased infiltration and cytotoxicity of in C5a (+) tumors
Corrales et al. 76	Lung	C5a sustains MDSC population	–	–	–
Caso et al. 20	Mammary	–	–	Tumor-bearing mice show expansion of C3a- and C5aR-overexpressing monocytes with downregulated MHC II	–
Fuenmayor et al. 78	Breast	–	–	–	–
Markiewski et al. 2	Cervical	MDSCs express C5aR; C5a attracts MDSCs (primarily PMN-MDSCs) to tumor site; C5aR antagonism restricts MDSCs to tumor periphery, neutralizes MDSC function, and diminishes ROS/RNS generation in MO-MDSCs	–	–	–

C5aR, C5a receptor; MDSC, myeloid-derived suppressor cell; Treg, regulatory T cell; NK, natural killer; MHC, major histocompatibility complex; PMN, polymorphonuclear; MO, mononuclear; ROS, reactive oxygen species; RNS, reactive nitrogen species.

**Table 2:** Summary of evidence implicating the complement anaphylatoxins as regulators of CD4+ and CD8+ T cells, B cells, granulocytes, and cytokine production in experimental cancer models

Study	Cancer Model	CD4+/CD8+ T cells	B cells	Granulocytes	Cytokines	Comments
Nunez et al. 74	Ovarian	CD8+ T cells increased by partial C3 deficiency	Unchanged by C3 deficiency	–	Cytokine production by macrophages, T cells, and B cells increased by partial C3 deficiency	Tumor immune infiltrate unchanged by C5aR deficiency
Gunn et al. 75	Lymphoma	CD4+ and CD8+ T cells (tumor and lymphoid organs) unchanged by C5a overexpression; C5a mediates Th1 differentiation	–	–	TNF-*α* production by macrophages reduced in C5a (+) tumors	C5a increases tumor cytotoxicity of innate leukocytes
Corrales et al. 76	Lung	–	–	C5a sustains granulocytic population	C5a promotes production of immunosuppressive cytokines	–
Caso et al. 20	Mammary	CD8+ T cell infiltrate strongly enhanced by C5aR antagonism	–	–	–	–
Fuenmayor et al. 78	Breast	–	–	Anti-HER2/neu mAb fused with C5a or C5a_desArg_ facilitates PMN granulocyte chemotaxis; C5a_desArg_ fusion protein most efficiently increases PMN survival and activation	–	Anti-HER2/neu mAb fused with C5a or C5a_desArg_ limits IgG3 binding to Fc*γ*Rs and has direct tumoricidal effect
Markiewski et al. 2	Cervical	–	–	–	–	Proliferation, apoptosis, and angiogenesis unchanged by C5aR antagonism; larger, more proliferative splenic white pulp follicles with C5aR depletion

C5aR, C5a receptor; Th1, T helper 1; TNF, tumor necrosis factor; mAb, monoclonal antibody; PMN, polymorphonuclear; Fc*γ*R, Fc-receptor for IgG; HER2/neu, human epidermal growth factor receptor 2.

### In ovarian cancer

Nunez-Cruz et al. 74. found that mice with ovarian tumors partially or fully C3-deficient had a significantly different tumor immune infiltrate compared to controls. Partially deficient mice had more CD8+ T cells and fewer Treg cells, and immune cells extracted from these tumors had attenuated cytokine production upon stimulation (with lipopolysaccharide and IFN-*γ*, or anti-CD3 and anti-CD28) compared with controls. Specifically, there was less elaboration of IL-10 and IL-12 by macrophages, IL-10 by B cells, and IFN-*γ* by T cells in partially C3-deficient mice. However, C3-deficient ovarian tumors had similar levels of macrophages, B cells, and MDSCs in their microenvironment, and the overall percentage of tumor-infiltrating leukocytes across the groups was similar. Genetic C3 deficiency impaired ovarian tumor development and growth in this experimental model, whereas genetic C5aR deficiency neither modified the tumor immune infiltrate nor affected tumor size compared with partially deficient mice.

### In lymphoma

Gunn et al. 75. found that C5a-expressing tumors had significantly increased infiltration of macrophages and NK cells and lower TNF-*α* production. C5a also increased the vulnerability of neoplastic cells to cytotoxic attack by NK cells and neutrophils from naïve mice. High C5a-producing syngeneic lymphomas had decreased CD4+ and CD8+ T cells in the tumor microenvironment, tumor-draining lymph nodes, and spleen, along with more MDSCs in the spleen. Accordingly, high C5a-producing tumors had enhanced tumor progression. While the frequency of neutrophil-like MDSCs was unchanged, these cells were less suppressive when extracted from C5a-producing tumors. Mice bearing low C5a-producing lymphomas had amplification of IFN-*γ*-producing CD4+ and CD8+ T cells in tumor-draining lymph nodes and the spleen, along with significantly decreased tumor burden. C5a was found to mediate Th1 (as per IFN-*γ* production) and Treg cell differentiation in a concentration-dependent, bell-shaped fashion such that high C5a levels decreased Th1 and increased Treg differentiation.

### In lung cancer

Corrales et al. 76 demonstrated that lung cancer cell lines deposit C5 and release C5a to a greater extent than nonmalignant bronchial epithelial cells, even in the absence of serum. Tumors treated with a C5aR antagonist grew slower than controls. In corresponding fashion, patients with non-small cell lung cancer had significantly higher plasma C5a levels, suggesting a systemic role for this complement-activation product. C5a appeared to promote an immunosuppressive microenvironment, as C5aR antagonism attenuated the population of MDSCs, including the granulocytic subpopulation, and expression of several immunosuppressive molecules, most of which promote Treg activity 77: ARG1, CTLA4, IL-6, IL-10, LAG3, and PDL1. However, the C5aR antagonist-treated group had a comparable proportion of CD4+, CD8+, and Treg cells.

### In mammary cancer

Caso et al. 20 demonstrated that mice harboring mammary tumors have a ninefold expansion of circulating blood monocytes compared with controls. These monocytes, in turn, strongly overexpress C3 and C5aR relative to controls, as well as several molecules involved in inflammation and immunosuppression, while exhibiting reduced MHC II expression, a strategy by which tumors escape host immune defenses.

### In breast cancer

Fuenmayor et al. 78 demonstrated that the use of a mAb against human epidermal growth factor receptor 2 (HER2/neu) fused with either C5a or C5a_desArg_ decreased the survival of breast cancer cells through a direct tumoricidal effect, in contrast to the anti-HER2/neu mAb alone. These findings were reproduced in coculture with human peripheral blood leukocytes. These fusion proteins facilitated chemotaxis of human PMN granulocytes, which are the primary immune effector cell responsible for facilitating ADCC. The C5a_desArg_ fusion protein most efficiently increased PMN survival and activation, as indicated by expression of the integrin Mac-1. Lastly, both fusion proteins significantly limited binding of a human IgG3 to Fc-receptors for IgG (Fc*γ*Rs), which are acute inflammatory mediators that have a tumoricidal role in anti-HER2/neu mAb therapy. Fc*γ*Rs are expressed by cytotoxic leukocytes including cytokine-activated PMNs, monocytes, and macrophages 79.

### In cervical cancer

Markiewski et al. 2 showed that mice bearing cervical tumors had robust deposition of C3 cleavage products throughout the tumor vasculature, indicating complement activation, though plasma levels of circulating C3 cleavage products were similar compared with controls, suggesting that a primarily local phenomenon shapes the tumor microenvironment. Furthermore, complement proteins were abundant within tumors, implying that C5a was generated through local complement initiation. C3 deficiency and C5aR inhibition or deficiency hindered tumor growth. Remarkably, pharmacological complement blockade using a C5a peptide antagonist was equally effective at limiting tumor growth as the conventional chemotherapeutic drug paclitaxel. The authors underscored the immunomodulatory effects of complement in tumor growth by first establishing that C5aR signaling does not alter tumor cell proliferation, apoptosis, or angiogenesis. C5aR antagonism was found to strongly enhance the CD8+ T cell tumor infiltrate relative to controls. In turn, the quantity of infiltrating CD8+ cells was inversely associated with tumor size. There was a trend toward a higher proportion of activated CD8+ T cells in C5aR-depleted tumors compared with controls. This group also exhibited larger and more proliferative splenic white pulp follicles. Importantly, the antitumor effects of C5aR deficiency were completely abrogated upon dissipation of the CD8+ T cell population in these mice using anti-CD8 antibody, in a dose-dependent fashion, while the same manipulation had no effect on tumor growth in the control group.

In both tumor-bearing and naïve mice, splenic and circulating MDSCs were found to express C5aR, akin to mature granulocytes and monocytes. Tumor-associated MDSCs had lower surface C5aR expression due to apparent internalization of C5aR, which the authors attributed to overstimulation by its ligand in the tumor microenvironment. C5a, a potent chemoattractant, was implicated in MDSC migration into tumors. C5aR inhibition limited the distribution of MDSCs cells to the tumor periphery, compared with the diffuse distribution seen in controls. Furthermore, the quantity of MDSCs directly correlated with tumor volume. The percentage of MDSCs recovered from C5aR-deficient mice was lower than in wild-type mice, though not significantly, as were the percentage of splenic MDSCs and the ratio of polymorphonuclear MDSCs (PMN-MDSCs) to mononuclear MDSCs (MO-MDSCs), suggesting that C5a is primarily a chemoattractant for PMN-MDSCs.

Splenic and intratumoral MDSC expression of CD11b, an integrin subunit necessary for MDSC adhesion to endothelial cells and extrusion from the circulation into the interstitial tissue of the tumor, was upregulated in PMN-MDSCs but not MO-MDSCs following C5a treatment in vitro. This effect was abrogated by the use of C5aR-deficient MDSCs. Furthermore, MDSCs extracted from the tumor microenvironment of C5aR-deficient mice had a partially or completely diminished ability to modulate splenic T-cell proliferation ex vivo relative to controls, suggesting that C5a also enhances MDSC suppression of the antitumor T-cell response. Tumor-derived MO-MDSCs, but not PMN-MDSCs, generated much lower levels of immunosuppressive ROS and RNS in C5aR-deficient mice relative to controls both in vivo and in vitro. Expression of arginase-1, an enzyme that bolsters ROS and RNS production by MDSCs, was significantly associated with tumor volume, though C5aR inhibition only slightly reduced arginase-1 levels.

## Discussion

Complement has been traditionally considered an important defense against pathogens, other nonself elements and neoplastic cells. However, recent research has identified a role for the complement activation products C3a and C5a in the paradoxical promotion of tumor progression. As several types of immune effector cells within the tumor microenvironment express the cognate receptors for C3a and C5a, investigators have postulated that intratumoral elaboration of anaphylatoxins drives local immunosuppression and at least partially accounts for the newly recognized cancer-promoting role of complement activation 1,3–5.

The studies reviewed in this article investigated the relationship between local and/or systemic complement signaling, the host immune response, and tumor progression in experimental models of lymphoma and ovarian, mammary, breast, lung, and cervical cancer. Their findings collectively support the paradigm that C3aR and/or C5aR signaling modifies the immune infiltrate within the tumor microenvironment and/or the peripheral blood and lymphoid organs, with consequential effects on tumor growth. In addition, complement activation modulates the function or efficiency of several types of immune effector cells, albeit sometimes contradictorily, as demonstrated in the lymphoma model of Gunn et al. These include both innate and adaptive effectors of host immunosurveillance as well as the immunosuppressive, cancer-sustaining MDSCs and Treg cells. Findings from in vivo cancer models clearly indicate that tumor progression can be halted and tumor regression achieved through the restoration of effective antitumor immunity 80. Furthermore, the composition, distribution, and density of the tumor inflammatory infiltrate vary across patients with cancer and may be prognostic 81–83, although this is controversial 84–86. Thus, it is critical to identify pathways and molecular targets that shape and regulate the tumor immune microenvironment.

Further research is required to characterize C3aR and C5aR expression and interactions with complement in other types of immune effector cells including dendritic cells and CD4+ T cells. Future investigation should also clarify whether the immunomodulatory functions of complement are concentration-dependent, as the results of Gunn et al. suggest. Furthermore, the findings reviewed here must be recapitulated in other experimental models, extended from in vitro to in vivo models, and ultimately appraised in the context of human biology.

It remains unclear to what extent the complement anaphylatoxins promote cancer through immunomodulatory effects versus other mechanisms. In a recently published study, Nitta et al. 87. demonstrated that C5a enhances cytoskeletal rearrangement, motility, matrix metalloproteinase secretion, and invasiveness in vitro in several C5aR-expressing cancer cell lines, as well as invasiveness in vivo in a C5aR-expressing bile duct cancer model. The potential utility of the complement anaphylatoxins as cancer biomarkers should also be further explored. In contrast to Corrales et al., who demonstrated elevated levels of circulating C5a, Ornellas et al. 88 demonstrated that C3 and C4a/b fragments were downregulated in the plasma of patients with penile squamous cell carcinoma. These biomarkers had a very high sensitivity and specificity for lymphatic spread and mortality, indicating a close correlation between these fragments and disease progression. Of note, the same research group showed that the cytotoxicity of circulating NK cells is impaired in patients with penile cancer 89, suggesting a paradigm of weakened innate immunity in this disease. Thus, these conflicting findings must be reconciled in future studies.

It is also unclear how host-specific factors relating to immunity, such as chronic viral infections, affect the levels of complement factors in patients with cancer. It has been suggested that infection with human papillomavirus (HPV) and/or Epstein–Barr virus (EBV), which are prevalent in certain squamous cell carcinomas 90, may alter complement activity 88, given that viral proteins are known to impair the immune response 91. While viruses do not independently initiate cancer development, they may contribute to the multifactorial process of cancer progression either through oncogenic effects or inhibition of tumor-suppressor proteins, particularly in cervical cancer (HPV), Burkitt's lymphoma (EBV), hepatocellular carcinoma (hepatitis viruses), and T-cell leukemia (retroviruses) 92.

Moreover, the balance between beneficial and adverse functions of complement must be verified in the context of cancer therapeutics. For instance, reduction in complement resistance through blockade of mCRPs has been shown to enhance the efficacy of mAb immunotherapy, which requires effective ADCC and complement-dependent cytotoxicity 93–95. As reviewed here, Fuenmayor et al. demonstrated that fusion of an anti-HER2/neu mAb with either C5a or C5a_desArg_ decreased the survival of breast cancer cells. Conversely, the findings of the other studies discussed here suggest that pathological complement activation is a desirable therapeutic target. Despite the evidence favoring this new paradigm of complement anaphylatoxin-supported tumor progression under defined experimental conditions, it is premature to infer that targeting C3aR and C5aR would be an appropriate adjuvant antitumor strategy. It has been speculated that C3aR or C5aR antagonists may be useful in cancer immunotherapy as adjuvants to vaccine-based approaches in patients with adverse prognoses, with the benefit of lower treatment toxicity than conventional cytotoxic chemotherapy 1. It may be desirable for future treatment strategies to target multiple complement-related genes in addition to cell survival and growth genes, ideally by identifying a shared transcription factor involved in their control 36.

## Conflict of Interest

The authors have no conflicts of interest to report. This work was supported by grants from the Howard Hughes Medical Institute (ETS), the Reza and Georgianna Khatib Endowed Chair in Skull Base Tumor Surgery at UCSF (ATP), and the Michael J. Marchese Professor and Chair at Northwestern University (ATP).
